# The External Use of Tincture of Belladonna in Night Sweating

**Published:** 1878-06

**Authors:** 


The External Use of Tincture of Belladonna in Night-Sweating.—Mr. Nairne writes, in the *British Medical Journal* of February 2, that for some little time past he has employed the common pharmacopœial tincture of belladonna for sponging the body in cases of phthisical and excessive sweating, and invariably with marked benefit. So far as his experience goes, he has found it much better than anything else; if applied before a sweating comes on, it prevents it; if during the sweating, it almost immediately controls it. Two teaspoonfuls of the tincture, mixed with an equal quantity of whisky, are quite sufficient (applied with the hand) to cover the whole body and produce the desired effect.—*Buffalo Med. & Surg. Jour.*

